# Neurological manifestations in patients with COVID‐19: A systematic review and meta‐analysis

**DOI:** 10.1002/jcla.24403

**Published:** 2022-04-06

**Authors:** Marzie Mahdizade Ari, Mohamad Hosein Mohamadi, Negar Shadab Mehr, Sajjad Abbasimoghaddam, Amirhosein Shekartabar, Mohsen Heidary, Saeed Khoshnood

**Affiliations:** ^1^ 440827 Department of Microbiology School of Medicine Iran University of Medical Sciences Tehran Iran; ^2^ 56941 Microbial Biotechnology Research Centre Iran University of Medical Sciences Tehran Iran; ^3^ 56941 Student Research Committee Sabzevar University of Medical Sciences Sabzevar Iran; ^4^ 56941 Department of Laboratory Sciences School of Paramedical Sciences Sabzevar University of Medical Sciences Sabzevar Iran; ^5^ 56941 Cellular and Molecular Research Center Sabzevar University of Medical Sciences Sabzevar Iran; ^6^ Clinical Microbiology Research Center Ilam University of Medical Sciences Ilam Iran

**Keywords:** COVID‐19, nervous system, neurological manifestations, SARS‐CoV‐2

## Abstract

**Introduction:**

The intensification of coronavirus disease 2019 (COVID‐19) complications, severe symptoms, and high mortality rate has led researchers to focus on this significant issue. While respiratory and cardiac complications have been described as high‐risk manifestations in patients with COVID‐19, neurological complications can also enhance mortality. This study aimed to evaluate the prevalence of neurological complications arises from SARS‐CoV‐2 and assess the mortality rate from neurological complications.

**Material and Methods:**

Literature review was conducted by searching in PubMed/Medline, Web of Sciences, and Embase. After performing search strategies with relevant terms, a number of articles were excluded, including review articles, systematic review or meta‐analysis, duplicate publication of same researchers, congress abstracts, animal studies, case reports, case series, and articles reporting a history of neurological features prior to COVID‐19 infection. After retrieving the data, statistical analysis was performed using the STATA Version 14 software.

**Results:**

From 4455 retrieved publications, 20 articles were selected for further analysis. Among 18,258 included patients, 2791 showed neurological symptoms, which were classified into different groups. Headache, confusion, and fatigue were reported as the most non‐specific neurological features in confirmed COVID‐19 patients. Psychiatric symptoms, CNS disorders, cerebrovascular disorders, CNS inflammatory disorders, PNS disorders, neuromuscular disorders, etc., were defined as specific neurological manifestations. The pooled prevalence of neurological manifestations and mortality rate of COVID‐19 patients with neurological features were estimated to be 23.0% (95% CI: 17.8–29.2) and 29.1% (95% CI: 20.3–39.8), respectively.

**Conclusion:**

Neurological manifestations may commonly happen in patients with COVID‐19. This study reported a high prevalence of neurological complications and mortality rates in COVID‐19 patients. Therefore, patients with COVID‐19 who indicated neurological symptoms should be taken seriously and should receive early treatment to prevent undesirable events.

## Graphical abstract

A total of 4455 studies were collected from PubMed/Medline, Embase, and Web of Science databases. After the removal of duplicates, articles were screened for title and abstract, and 119 papers met the criteria. Thereafter, the full‐text of these studies, which all reported the neurological symptoms of COVID‐19 infection, were evaluated. Following the exclusion of irrelevant studies, 20 articles were identified as qualified for final extraction and analysis.

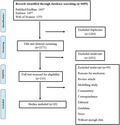

## 1 INTRODUCTION

The coronavirus disease 2019 (COVID‐19), an infection induced by severe acute respiratory syndrome coronavirus 2 (SARS‐COV‐2), originated in Wuhan, China in December 2019. This disease spread rapidly around the world and caused the deaths of millions of people.^1^ In 2002 and 2012, the outbreaks of other members of this betacoronavirus, namely SARS‐CoV and MERS‐CoV, were, respectively, recorded.^2^ According to the WHO report, more than 100 million cases were infected with SARS‐CoV‐2, and about four million deaths from COVID‐19 were reported.

Conforming to the Centers for Disease Control and Prevention (CDC), COVID‐19 is accompanied by three main symptoms, including common (fever, dry cough, headache, and fatigue), less common (pain, sore throat, diarrhea, and loss of taste and appetite), and severe (shortness of breath, chest pain, and dysfunction in speech). The disease is not limited to the respiratory system, but it encompasses a wide range of cardiovascular and neurological complications.^3^ Search of genomes and coronavirus‐related sequences in the cerebrospinal fluid (CSF) verifies this assumption.^4^ The intensified complications, severe symptoms, and high mortality rate of COVID‐19 have forced special attention to this disease. While respiratory and cardiac complications are considered high risks in patients with COVID‐19, neurological complications have also been demonstrated to enhance mortality rate.^5^

SARS‐CoV‐2, the same as SARS‐CoV and MERS‐CoV viruses, has several routes of entry to central nervous system (CNS) and peripheral nervous system (PNS), including olfactory pathway and gut–brain axis as the neural pathways^6^ and blood–brain barrier (BBB) as hematogenous pathway.^7^, ^8^ These accessibilities are provided by the expansion and expression of angiotensin‐converting enzyme 2 (ACE2) on nerve tissues and capillary endothelial tissues,^9^ which are also present in brain endothelial cells and small intestine.^10^ This notion corroborates earlier investigations that stated reduced RNA tracking in CSF may be the indication of direct SARS‐CoV‐2 invasion to nervous tissue in the cell‐to‐cell spread pattern.^11^, ^12^ However, inaccessibility to virus in the normal range of detection via the analysis of CSF samples and also the presence of hemoproteins product may relate to the lack of identifying SARS‐CoV‐2 in these samples.^13^

The first step in brain damage caused by the SARS‐COV‐2 is triggering the inflammatory response of cytokines (e.g., IL‐6, IL‐12, and TNF‐α) after binding the virus to ACE2 and its proliferation in the CNS.^5^, ^10^ Neurological complications that affect CNS, PNS, and musculoskeletal system^8^, ^13^ entail impaired consciousness, stroke, anosmia, ataxia, and seizures, acute necrotizing encephalopathy, meningoencephalitis, and Guillain–Barre syndrome (GBS), recognized as PNS‐related symptoms, as well as Miller Fisher syndrome and hypogeusia that happen as a result of SARS‐CoV‐2 entry to peripheral neurons.^14^ Collectively, the direct invasion of virus, immune‐induced complications, and cytokine storms are the main pathogenesis of the SARS‐CoV‐2. It is surmised that the immune system role is central to the development of neurological complications caused by this virus.^15^, ^16^ The present study aimed to evaluate the prevalence of neurological manifestations and mortality rate in patients with COVID‐19.

## 2 MATERIALS AND METHODS

The present systematic review was conducted by relying on Preferred Reporting Items for Systematic Reviews and Meta‐Analyses (PRISMA) statements.^17^

### 2.1 Search strategy

Our systematic search was performed using databases viz PubMed/Medline, Web of Sciences, and Embase from January 1, 2019 to March 7, 2021. The terms employed in the search strategies were as follows: “Central nervous system” or “brain” or “neurologic” or “CNS “and “COVID‐19” or “severe acute respiratory syndrome coronavirus 2” or “novel coronavirus” or “SARS‐CoV‐2” or “SARS2” or”2019‐nCoV” or “nCoV disease” or “coronavirus disease‐19” or “coronavirus disease 2019” or “2019 novel coronavirus” or “Wuhan coronavirus” or “Wuhan seafood market pneumonia virus” or “Wuhan pneumonia”. There were no language restrictions; however, for non‐English articles, online translation systems were utilized. Besides, no limitations were considered for the location and type of articles.

### 2.2 Inclusion and exclusion criteria

All original studies presenting the neurological symptoms in COVID‐19 patients were regarded in our search. The results of final search were imported into EndNote X9 software (Thomson Reuters, San Francisco, CA), and duplicates were eliminated. We performed a three‐step screening to determine eligible results based on title, abstract, and full‐text. In all the studies included, patients developed neurological features and were positive for SARS‐CoV‐2. Reverse transcription‐polymerase chain reaction (RT‐PCR) was the main diagnosis method of confirming COVID‐19, but multiple studies added serological tests and computed tomography (CT) scan, as well. Regarding nervous system involvement, we took into consideration all reported neurological findings, comprising CNS and PNS symptoms, CNS inflammatory features, and cerebrovascular attributes, as well as neuromuscular, psychiatric, and non‐specific symptoms. All the methods used for the diagnosis of neurological symptoms were viewed as qualified. The exclusion of articles was based on any of the following papers: Review articles, systematic review or meta‐analysis, duplicate publication of same researchers, congress abstracts, and articles reporting a history of neurological features prior to COVID‐19 infection. Likewise, animal studies, case reports, and case series were excluded.

### 2.3 Data extraction and quality assessment

The data retrieved for each article were as follows: first author's name, location, publication time, sample size, number of COVID‐19 patients, gender, median age, COVID‐19 diagnosis method, neurological manifestations, diagnosis method of neurological findings, and number of deaths. Four authors extracted the information from the full‐text of 20 selected studies, independently. Inconsistencies between reviewers were resolved by consultation. Table 1 shows the extracted data.

**TABLE 1:** Characteristics of the included studies

References	Country	Published time	No. of patients with COVID‐19	Male	Median age (years)	Covid‐19 diagnosis method	No. of patients with neurological manifestations	Diagnosis method for neurological manifestation	Neurological manifestations	Death
Helms^18^	France	Aug, 2020	140	100	62	RT‐PCR, CT scan	118	CNE, RASS, CAM‐ICU, EEG, MRI, CSF analysis	NM	21
Karadaş^8^	Turkey	Jun, 2020	239	133	39	RT‐PCR, CT scan	83	CNE, Brain CT, MRI, EEG	Headache 64, neuralgia 19, visual impairment 11, nizziness 16, auditory dysfunction 8, Numbness 12, Bifurcation in voice 3, Anosmia/hyposmia 18, Ageusia/dysgeusia 16, CVD 9, Impaired consciousness‐confusion 23, Sleep impairment 30, Orthostatic hypertension 8, Balance disorder 6, Myalgia 36, GBS 1, RLS 4	NM
Frontera^19^	USA	Jan, 2021	4491	2607	NM	RT‐PCR	606	CNE, Brain CT, MRI, EEG, CSF analysis	Encephalopathy 309, Stroke 84, Seizure 74, Hypoxic/ischemic brain injury 65, Movement disorder 41, Neuropathy 35, Myopathy 21, GBS 3	211
Luigetti^20^	Italy	Jul, 2020	213	137	70.2	RT‐PCR	64	CNE, CSF analysis	Headache 10, Dizziness 3, Balance disorder 3, Encephalopathy 86, Ageusia/dysgeusia 6, Anosmia/hyposmia 13, Seizure 6, Stroke 4, Encephalitis 1, Weakness 69, Myalgia 20, skeletal muscle injury 10	40
Kandemirli^21^	Turkey	Oct, 2020	235	NM	NM	RT‐PCR	50	CNE, MRI, CSF analysis	NM	NM
Sandoval^22^	Chile	Mar, 2021	90	NM	NM	RT‐PCR, ELISA	13	CNE, EEG, Brain CT, MRI, CSF analysis	Seizure 3, Encephalopathy 6, myalgia 8, Anosmia/hyposmia 2, Other cranial nerves impairments 2, orthostatic intolerance 2	3
Studart‐neto^53^	Brazil	Aug, 2020	1208	NM	57.4	RT‐PCR, CT scan	89	CNE, Brain CT, MRI, CSF analysis	Encephalopathy 43, Stroke 11, Cerebral venous Thrombosis 2, Intracranial hemorrhage 2, Seizure 8, neuropathy 3, Rhabdomyolysis 2, headache 3, vertigo 2, movement disorder 6, impaired consciousness 35, psychomotor agitation 12, delayed awakening from sedation 5, focal neurological deficit 3, weakness 16	14
Xiong^24^	China	Sep, 2020	917	504	NM	RT‐PCR, CT scan	39	CNE, CSF analysis, Brain CT	Delirium 7, coma 14, Syncope 3, Stroke 1, Myalgia 2, headache 2, neuralgia 1	30
Liguori^33^	Italy	Aug, 2020	103	59	55	RT‐PCR	94	CNE	Sleep impairment 51, Ageusia/dysgeusia 48, headache 40, Anosmia/hyposmia 40, depression 39, Auditory Dysfunction 2, Confusion 23, Dizziness 27, Numbness/Paresthesia 6, Fatigue 33, daytime sleepiness 34, Myalgia 25, Anxiety 34	NM
Scullen^25^	USA	Sep, 2020	76	40	59.8	RT‐PCR, CT scan	27	CNE, EEG, Brain CT, MRI, SWI	Altered mental status 26, Encephalopathy 22, vasculopathy 5, Ageusia/dysgeusia 1, Weakness 1, Headache 2, visual impairment 14, Decerebrate posturing 1, Facial droop 1, Hemineglect 2, Hemiparesis or hemiplegia 4, Quadriplegia 1	NM
Iltaf‐Sr^4^	Pakistan	Aug, 2020	350	245	49.5	RT‐PCR	68	CNE	Headache 12, vertigo 12, numbness/paresthesia 11, consciousness 7, Anosmia/hyposmia 5, encephalitis 3, Stroke 2, GBS 1, Seizure 1	NM
Khedr^26^	Egypt	Feb, 2021	439	NM	55.1	RT‐PCR, CT scan	117	CNE, Brain CT, MRI, EMG	Headache 47, dizziness 50, myalgia 40, anxiety 10, suicidal trial 1, CVD 55, convulsions 5, encephalitis 6, Encephalopathy 4, attack of relapse of RR‐MS 2, transverse myelitis 2, meningoencephalitits 1, GBS 4, neuropathy 3, myasthenia gravis 2, myositis 2, isolated cranial nerve affection 31, Anosmia/hyposmia 31	NM
Chougar^10^	France	Jul, 2020	1176	NM	61.2	RT‐PCR, CT scan	223	CNE, MRI, EEG, CSF analysis	Focal neurological deficit 43, Seizure 13, altered mental status 21, headache 31, confusion 30, impaired consciousness 40, coma 3, delayed awakening from sedation 11, peripheral vestibular syndrome 1, Anosmia/hyposmia 22, visual impairment 6, GBS 1	NM
Nersesjan^11^	Denmark	Jan, 2021	61	38	62.7	RT‐PCR	28	CNE, Brain CT, MRI, EEG, CSF analysis, NCS	Weakness 21, Anosmia/hyposmia 18, headache 10, sensory symptoms 3, Seizure 4, hallucination 12, affect lability 2, paranoia 4, delirium 21, Stroke 4, Encephalopathy 19, peripheral facial palsy 2, myalgia 2, encephalitis 2, myelitis 1, rhabdomyolysis 1, neuropathy 1, altered mental status 18, dysexecutive function 20, delayed awakening from sedation 9, coma 2	12
Abled‐Mannan^27^	UK	Oct, 2020	50	NM	11.7	RT‐PCR, ELISA	4	CNE, Brain CT, MRI, CSF analysis, EMG	Encephalopathy 4, headache 3, dysarthria or dysphagia 2, meningism 1, ataxia 1, myalgia 4, reduced reflexes 2	0
LaRovere^28^	USA	Feb, 2021	1695	909	9.1	RT‐PCR, ELISA	365	CNE, Brain CT, MRI, SWI	Encephalopathy 15, Stroke 12, GBS 4, acute CNS infection/ADEM 8, acute fulminant cerebral edema 4	11
Rifino^54^	Italy	Oct, 2020	1760	1162	64.9	RT‐PCR, CT scan	137	CNE, EEG, EP, ENG‐EMG, Brain CT, MRI, CSF analysis	CVD 53, Stroke 48, Transient ischemic attacks 4, Cerebral venous thrombosis 1, neuropathy 45, GBS 17, Altered mental status 49, Encephalitis 5, Myelitis 2, Headache 3, Seizure 10, Syncope 3, Movement disorder 7	41
Mao^30^	China	Apr, 2020	214	87	52.7	RT‐PCR, CT scan	78	CNE, Brain CT	Dizziness 36, Headache 28, Impaired consciousness 16, CVD 6, Ataxia 1, Seizure 1, ageusia/dysageusia 12, Anosmia/hyposmia 11, visual impairment 3, Neuralgia 5, Myalgia 23	NM
Eskandar^31^	Montefiore	Dec, 2020	4711	NM	63.4	RT‐PCR	581	CNE, Brain CT, MRI	Altered mental status 258, Stroke 55, seizures 26	199
Shekhar^32^	USA	Aug, 2020	90	NM	52.3	RT‐PCR	7	CNE, MRI, EEG, Brain CT, DWI	Altered mental status 7, visual impirment 2, Seizure 4	2

Abbreviations: CNE, clinical neurological exams; CAM‐ICU, confusion assessment method for the ICU; CT, computerized tomography; CVD, cerebrovascular disease; EEG, electroencephalography; EMG, electromyography; ENG‐EMG, electroneurographic and electromyographic recordings; EP, evoked potentials; GBS: Guillain–Barre syndrome; MRI, magnetic resonance imaging; NCS, nerve conduction study; NM, not mentioned; RASS, Richmond Agitation‐Sedation Scale; RLS, restless leg syndrome; SWI, susceptibility weighted imaging.

### 2.4 Data synthesis and analysis

Analysis of data was performed by using STATA (version 14, IC; Stata Corporation, College Station, TX, USA), and the prevalence of neurological complications was estimated with confidence intervals (CIs) of 95%. The pooled frequency was calculated by a random effect model with 95% CI. Publication bias was also assessed by Begg's and Egger's tests, and *p* value <0.05 was considered an indication of statistically significant publication bias.

## 3 RESULTS

**FIGURE 1:**
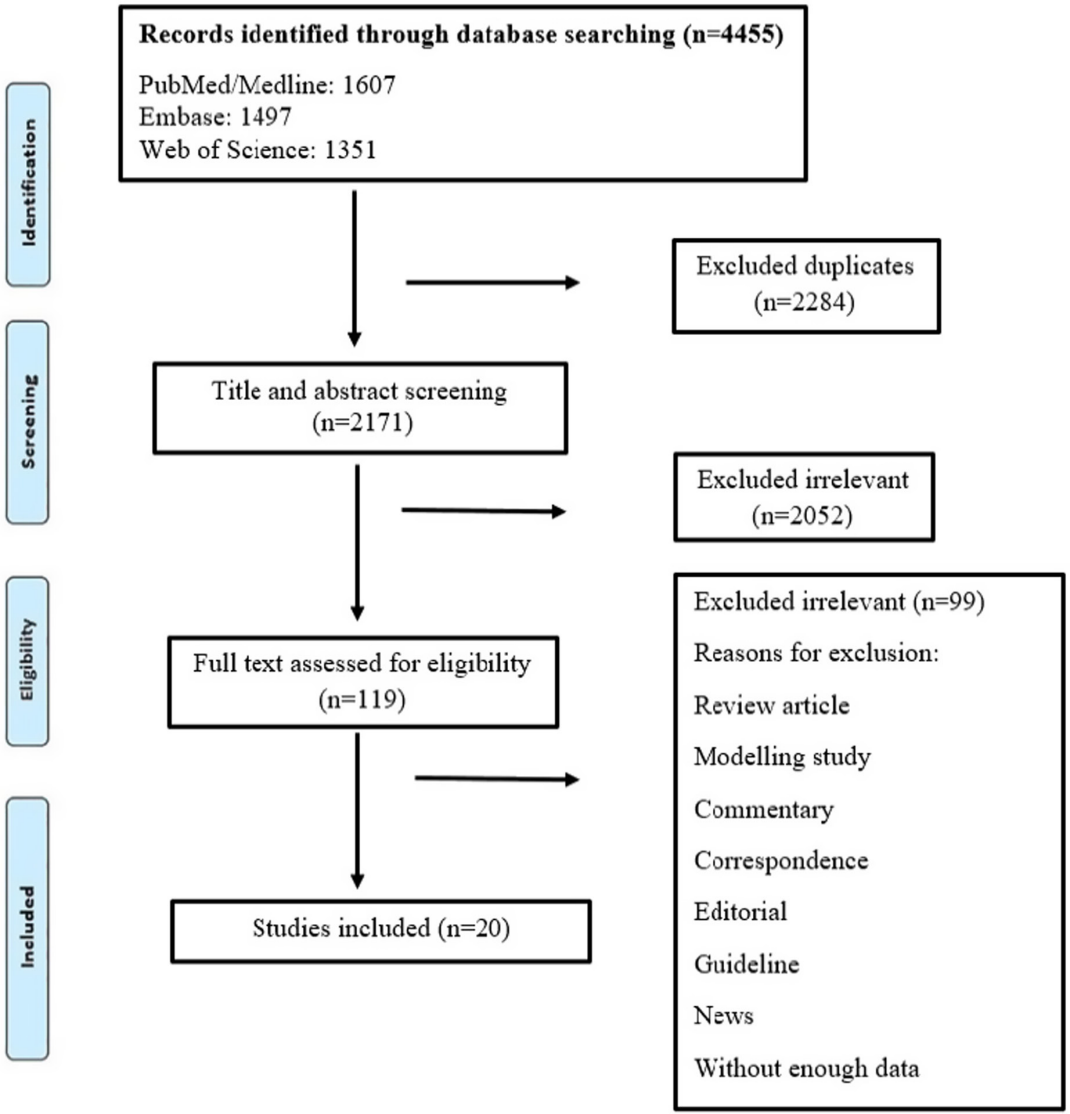
Flow diagram detailing review process and study selection

A total of 4455 studies were collected from three databases. After the removal of duplicates, articles were screened for title and abstract, and 119 papers met the criteria. Thereafter, the full‐text of these studies, which all reported the neurological symptoms of COVID‐19 infection, were evaluated. Following the exclusion of irrelevant studies, only 20 articles were identified as qualified for final extraction and analysis (Figure 1). These papers were appropriate for systematic review and entered into data extraction. Eighteen of these clinical studies used various methods, such as brain CT, MRI, EGG, and CSF for the diagnosis of neurological manifestations.^8^, ^10^, ^11^, ^18^, ^19^, ^20^, ^21^, ^22^, ^23^, ^24^, ^25^, ^26^, ^27^, ^28^, ^29^, ^30^, ^31^, ^32^ However, two other studies performed only clinical neurological exams.^4^, ^33^ In all 20 studies, RT‐PCR was examined using nasopharyngeal swab, but nine^8^, ^10^, ^18^, ^23^, ^24^, ^25^, ^26^, ^29^, ^30^ and three^22^, ^27^, ^28^ studies employed chest CT scan and ELISA, respectively, to confirm COVID‐19. Ten articles were originated from Europe, five from the USA, two from China, and others from Brazil, Pakistan, Egypt, and Chile. The information of these 20 studies is summarized in Table 1. A variety of neurological findings were reviewed and classified into different groups (Table 2). Among 20 articles, 18,258 cases were found by RT‐PCR test to be positive for SARS‐CoV‐2 (COVID‐19), and 2791 of patients had various neurological manifestations. The overall frequency of neurological symptoms in COVID‐19 patients was 15.28%. Additionally, 12 articles reported mortality. Among the 2791 patients, 582 succumbed in hospital; therefore, the total mortality rate was 20.85%.

**TABLE 2:** Categorized neurological findings of included studies

Variables	No. of studies	*n*/*N*	Percentage (%)
Non‐specific symptoms			
Headache	13	255/1051	24.26
Dizziness			
Weakness	5	132/436	30.27
Fatigue	3	38/144	26.39
Impaired consciousness/confusion	1	33/94	35.10
Syncope	6	174/635	27.40
Neuropathy	2	6/176	3.40
Altered mental status	5	87/977	8.81
Neuralgia	6	379/1003	37.78
Sleep impairment	3	25/200	12.50
Daytime sleepiness	2	81/177	45.76
Bifurcation in voice	1	34/94	36.17
Balance disorder	1	3/83	3.61
Numbness/paresthesia	2	10/177	5.65
Vertigo	3	29/245	11.83
Reduced reflexes	2	14/157	8.92
Hemiparesis/hemiplegia	1	2/4	50
Quadriplegia	1	4/27	14.81
Peripheral facial palsy	1	1/27	3.70
Hemineglect	1	2/28	7.14
Focal neurological deficit	1	2/27	7.40%
	2	46/312	14.74%
Psychiatric symptoms			
Depression	1	39/94	41.50%
Anxiety	2	44/211	20.85%
Delirium	2	28/67	41.79%
Paranoia	1	4/28	14.28%
Hallucination	1	12/28	42.86%
Suicidal trial	1	1/117	0.85%
Affect lability	1	2/28	7.14%
Psychomotor agitation	1	12/89	13.48%
CNS disorders			
Seizure/convulsion	12	155/2011	7.71
Stroke	9	221/1977	11.18
Coma	3	19/290	6.55
Meningism	1	1/4	25
Isolated cranial nerve affection	1	31/117	26.49
Dysexecutive function	1	20/28	71.43
Orthostatic hypertension	1	8/83	9.64
Orthostatic intolerance	1	2/13	15.38
Ataxia	2	2/82	2.44
Acute fulminant cerebral edema	1	4/365	1.09
Other cranial nerve impairments	1	2/13	15.38
Cerebrovascular disorders			
Cerebral venous thrombosis	2	3/226	1.33
Transient ischemic attacks	1	4/137	2.92
Intracranial hemorrhage	1	2/89	2.25
CVD	4	123/415	29.64
Vasculopathy	1	5/27	18.52
Hypoxic/ischemic brain injury	1	65/606	10.72
CNS inflammatory disorders			
Encephalopathy	8	422/1249	33.79
Encephalitis	5	17/414	4.10
Transverse myelitis	1	2/117	1.71
Myelitis	2	3/165	1.82
Meningoencephalitis	1	1/117	0.85
GBS	7	31/1599	1.94
Acute CNS infection/ADEM	1	8/365	2.19
PNS disorders			
Anosmia/hyposmia	9	160/768	20.83
Ageusia/dysageusia	5	83/346	23.99
Auditory dysfunction	2	10/177	5.65
Visual impairment	5	36/418	8.61
Peripheral vestibular syndrome	1	1/223	0.45
Sensory symptoms	1	3/28	10.71
Myasthenia gravis	1	2/117	1.70
Attack of relapse of RR‐MS	1	2/117	1.70
RLS	1	4/83	4.82
Neuromuscular disorders			
Myalgia	9	139/520	26.73
Myopathy	1	21/606	3.46
Movement disorder	3	54/832	6.49
Myositis	1	2/117	1.70
Dysarthria/dysphagia	1	2/4	50
Rhabdomyolysis	2	3/117	2.56
Facial droop	1	1/27	3.70
Decerebrate posturing	1	1/27	3.70
Skeletal muscle injury	1	10/64	15.62
Others			
Delayed awakening from sedation	3	25/340	7.35

### 3.1 Non‐specific neurological features in confirmed COVID‐19 patients

Since the emerging COVID‐19 infection, a noticeable large number of studies have focused their attention on this outstanding issue; thus, various symptoms and signs have been reported for this disease. Based on the literature, headache,^34^ confusion,^35^ and fatigue^36^, ^37^ are the most common symptoms. In addition, altered mental status is realized as a common initial presentation in COVID‐19 patients.^38^ Our results are in line with these findings. Among 20 included studies we reviewed, 13, 5, 3, 1, 6, and 6 studies reported headache, dizziness, weakness, fatigue, confusion, and altered mental status, respectively, as the most frequent symptoms. Five studies reported the observation of neuropathy features in 87 of 977 examined patients.^11^, ^19^, ^23^, ^26^, ^29^ Two articles stated 45.76% (81/177) with sleep disorder,^8^, ^33^ and one of these studies mentioned daytime sleepiness in 36.17% (34/94).^33^ The relative frequency of reduced reflex symptoms was 50% (2/4), standing as the most common symptoms. However, it could not be considered a notable manifestation due to the inadequate number of cases.^27^ Hemiparesis and hemiplegia were detected in 14.81% (4/27), as reported by one study.^25^

### 3.2 Characteristics of CNS disorders in confirmed COVID‐19 patients

Evidence has shown that coronavirus can target CNS and exert its neurotropic effects.^39^ On the basis of this review, a broad spectrum of CNS manifestations was reported in coronavirus‐infected patients and classified into CNS disorders, CNS inflammatory disorders, and cerebrovascular disorders. The number of stroke (11.18%), coma (6.55%), and seizure/convulsion (7.71%) patients were much more than any other CNS disorders. Thirteen studies reported at least one of these disorders in COVID‐19 patients.^4^, ^10^, ^11^, ^19^, ^20^, ^22^, ^23^, ^24^, ^29^, ^30^, ^31^, ^32^ Remarkably, stroke, as a major concern in COVID‐19, was identified in an overall number of 211 out of 1977 patients. Neuroinvasion of coronavirus can disrupt immune system of CNS, leading to CNS inflammatory disorder.^40^, ^41^ Our results identified encephalopathy,^11^, ^19^, ^20^, ^22^, ^23^, ^25^, ^26^, ^27^, ^28^ encephalitis,^4^, ^11^, ^20^, ^26^, ^29^ transverse myelitis,^26^ meningoencephalitis,^26^ and GBS^4^, ^8^, ^10^, ^19^, ^26^, ^28^, ^29^ as the inflammatory disorders of CNS. Among these disorders, encephalopathy had notable symptoms with a high frequency of 33.79% (422/1249). CNS and vascular system are coupled and inseparable systems influencing each other^42^; therefore, alteration of CNS can affect vascular systems. This review is consistent with the clinical symptoms of these effects. Cerebral venous thrombosis,^23^, ^29^ vasculopathy,^25^ and cardiovascular diseases^8^, ^26^, ^29^, ^30^ were prominent cerebrovascular manifestations of COVID‐19.

### 3.3 Characteristics of PNS and psychiatric symptoms in COVID‐19 patients

Nine studies reported 160 anosmia/hyposmia cases out of 768 COVID‐19 patients.^4^, ^8^, ^10^, ^11^, ^20^, ^22^, ^26^, ^30^, ^33^ Six articles emphasized at least one of the following features: ageusia/dysgeusia (160 patients), auditory dysfunction (10 patients), and visual impairment (36 patients). Myopathy^19^ and myalgia^8^, ^11^, ^20^, ^22^, ^24^, ^26^, ^27^, ^30^, ^33^ had the overall frequencies of 3.46% and 26.73%, respectively. Furthermore, some rare cases of rhabdomyolysis,^11^, ^23^ myositis,^26^ and dysarthria^27^ were explored. Coronavirus can affect nervous system in a way that develops psychiatric symptoms. Depression (41.50%), anxiety (20.85%), delirium (41.79%), and hallucination (42.86%) were behavioral features observed as the most common psychiatric manifestations.^11^, ^24^, ^26^, ^33^ A few cases of paranoia,^11^ suicidal trial,^26^ and psychomotor agitation^23^ were also reported by three articles.

### 3.4 Prevalence of neurological manifestations in COVID‐19 patients

**FIGURE 2:**
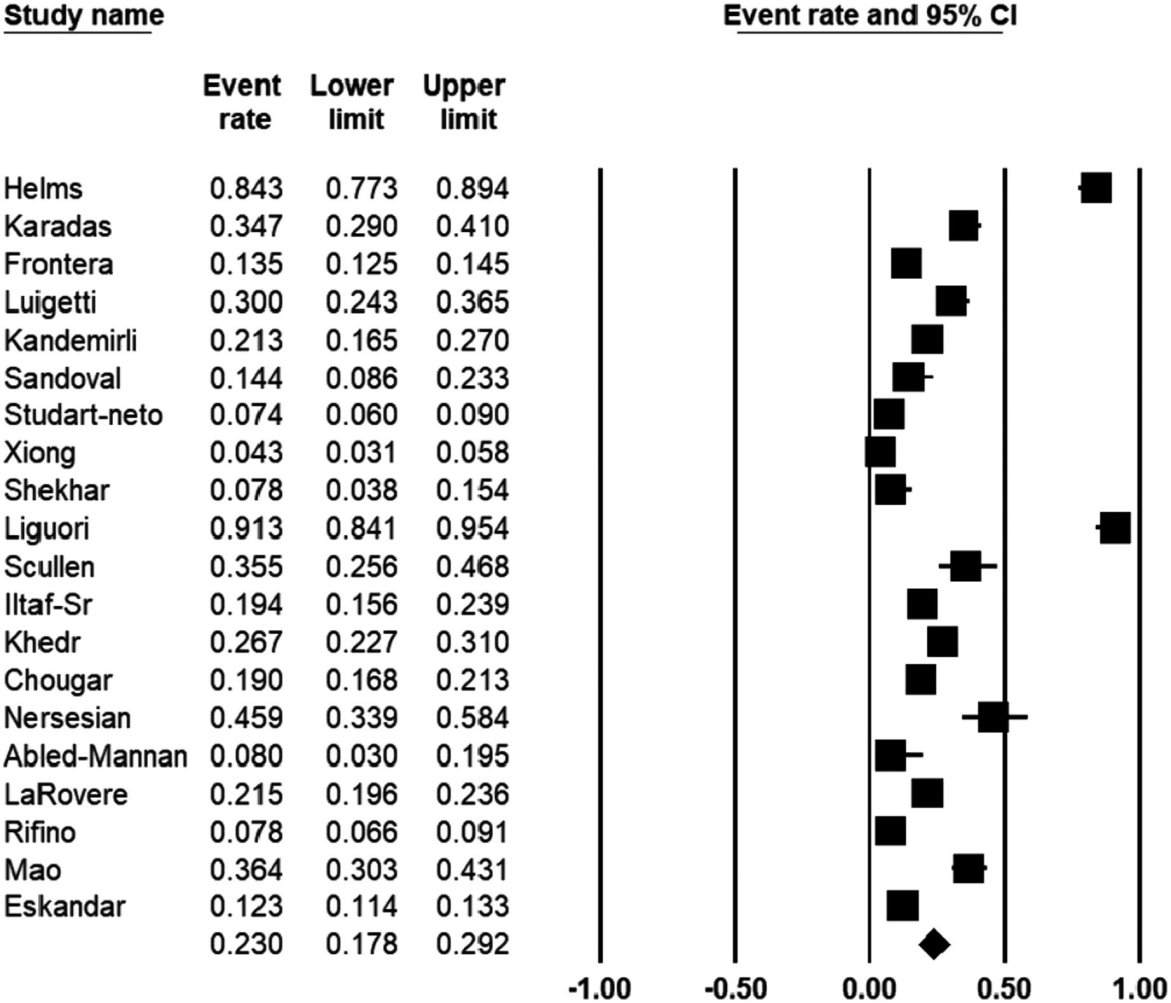
Forest plot of meta‐analysis on neurological manifestations of COVID‐19

**FIGURE 3:**
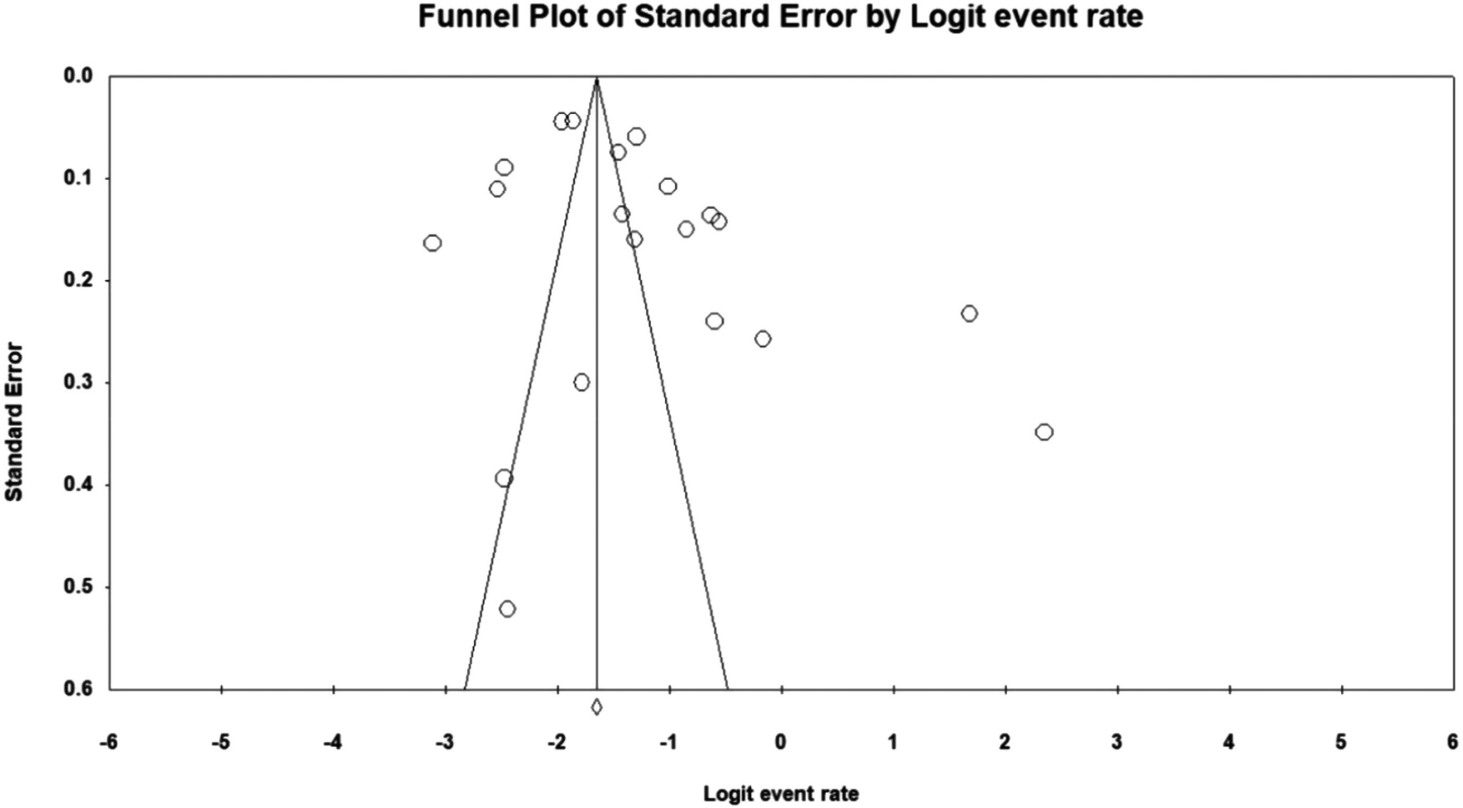
Funnel plot of meta‐analysis on neurological manifestations of COVID‐19

From 20 studies examined, the pooled prevalence of neurological manifestations was estimated as 23% (95% CI 17.8–29.2) in COVID‐19 patients. Figure 2 depicts a forest plot for meta‐analysis of nervous system involvement in COVID‐19. There was no publication bias (*p* = 0.54 for Begg's rank correlation analysis and *p* = 0.06 for Egger's weighted regression analysis), as shown in Figure 3.

### 3.5 Mortality of COVID‐19 patients who developed nervous system symptoms

**FIGURE 4:**
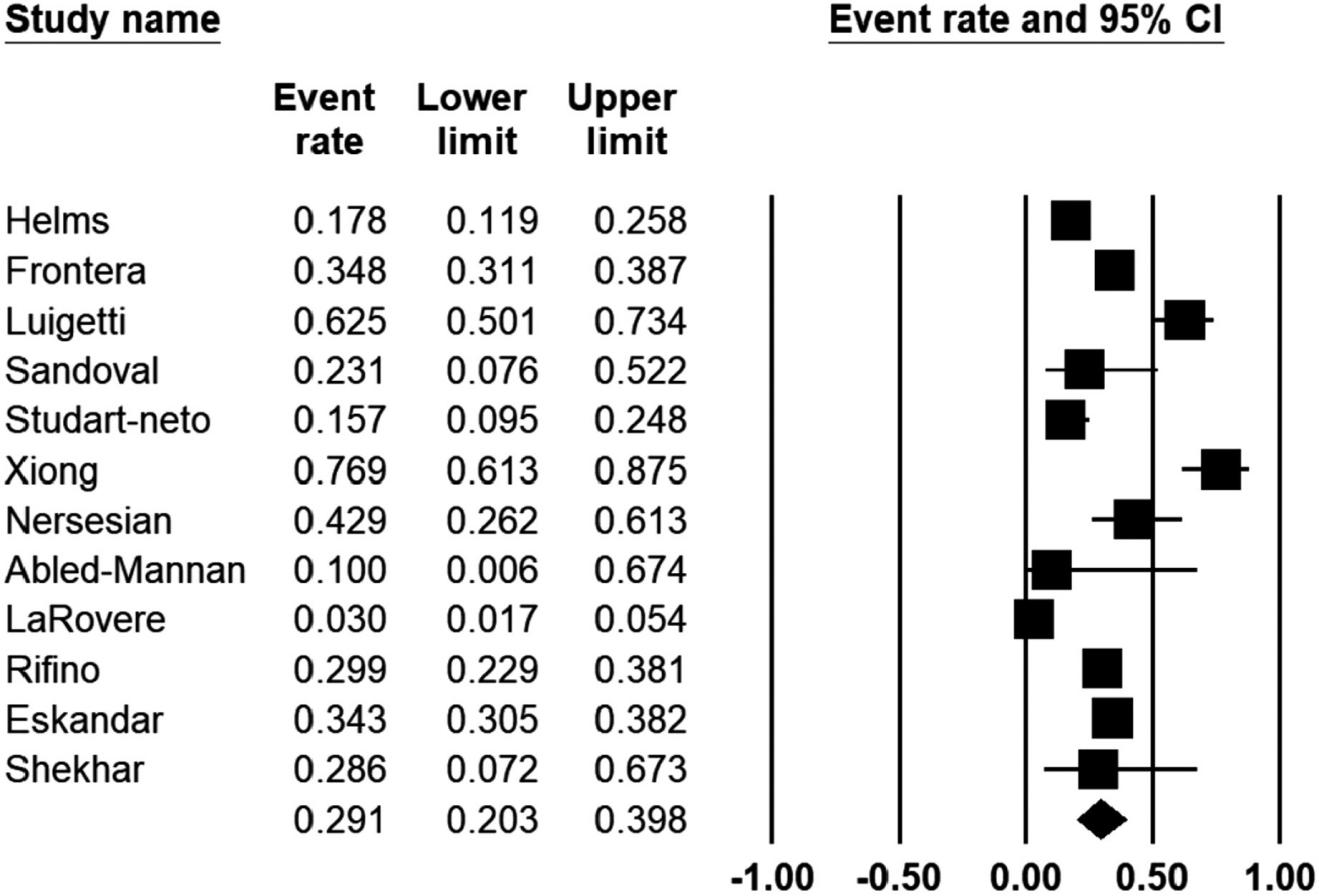
Forest plot of meta‐analysis on mortality rate from neurological features of COVID‐19

**FIGURE 5:**
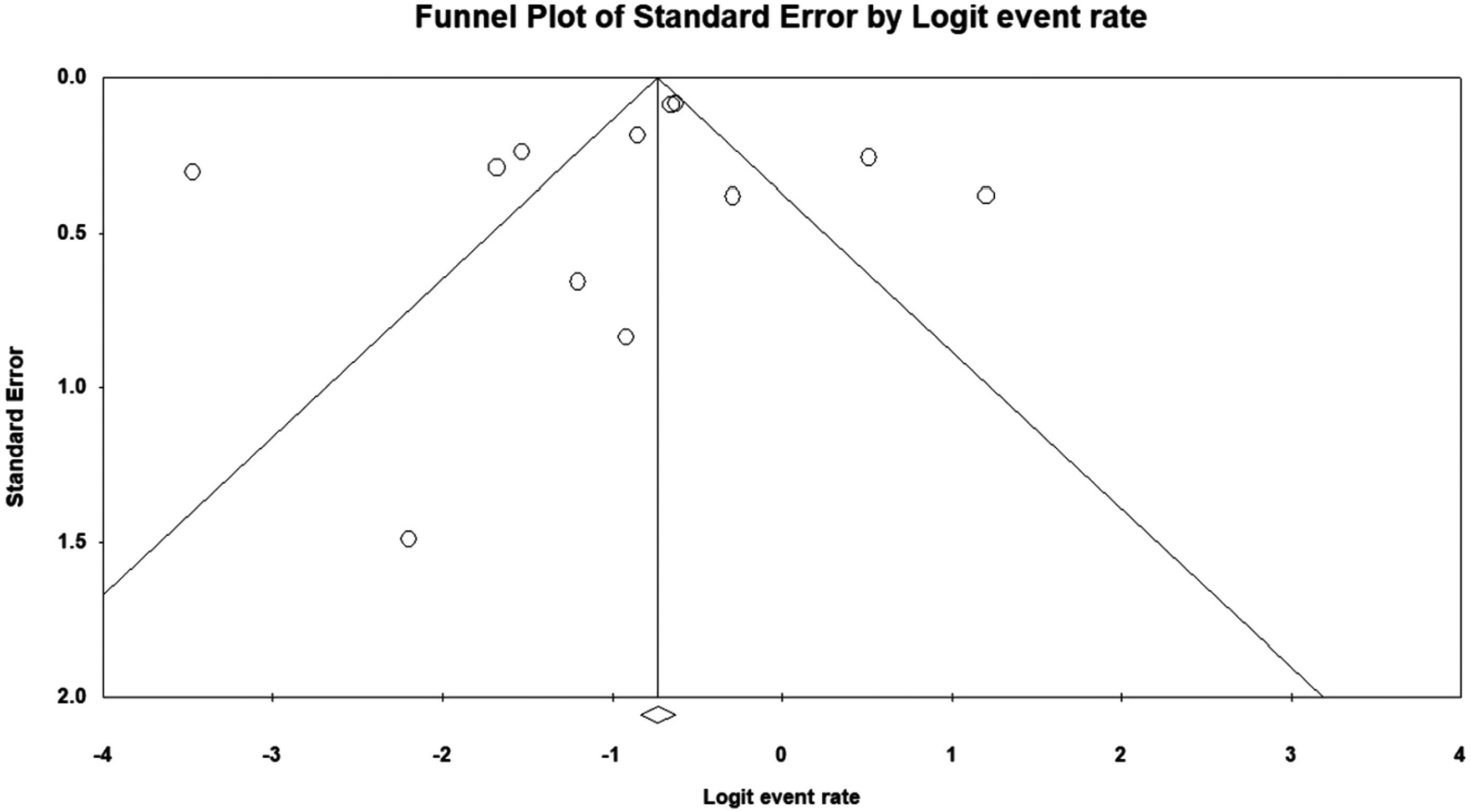
Funnel plot of meta‐analysis on the mortality rate from neurological features of COVID‐19

In total, 12 studies reported the number of deaths as the outcome. The pooled mortality of COVID‐19 patients with neurological features was estimated to be 29.1% (95% CI: 20.3–39.8). Figure 4 displays the meta‐analysis of the overall mortality rate. As indicated in Figure 5, no evidence of publication bias was found (*p* = 0.54 and *p* = 0.57 for Begg's rank correlation analysis and Egger's weighted regression analysis, respectively).

### 3.6 Risk of bias assessment

According to the quality assessment of the 20 included studies, all of them had a low risk of bias. We summarized the results of the critical appraisal (JBI checklist) of included studies in Table S1.

## 4 DISCUSSION

The present study was performed on 18,258 enrolled COVID‐19 patients, from which 2791 cases showed neurological complications. To the best of our knowledge, no survey has hitherto addressed the prevalence of neurological complications of COVID‐19, and this systematic review/meta‐analysis is the first study to investigate the mortality rate and prevalence of neurological manifestations, which is classified into several subgroups. In a study performed by Mao et al., the evaluation of neurological complications in COVID‐19 patients contributes to the early diagnosis of severe respiratory disease, prevention of death, and control of transmission cycle to other healthy individuals.^43^ Therefore, through the evaluation of COVID‐19‐induced neurological damages and underlying mechanisms, more effective treatment strategies can be proposed to decline the severity and impact of SARS‐CoV‐2 on the nervous system.^23^ The result of our study revealed that the overall prevalence of neurological complications of COVID‐19 is 23%. This high rate may have led to later recovery of patients with COVID‐19 and their longer hospital stay. Overexpression of ACE2 in nervous tissues and proinflammatory cytokines could be a major factor affecting vascular endothelium and elevating BBB permeability.^12^ Neurological manifestations of COVID‐19 are defined in two general groups: non‐specific and specific symptoms. Psychiatric symptoms, CNS disorders, cerebrovascular disorders, CNS inflammatory disorders, PNS disorders, and neuromuscular disorders are recognized as non‐specific and other symptoms as specific neurological manifestations. Non‐specific neurological complications can arise from systematic behaviors,^44^ whereas specific complications are associated with direct virus invasion to nervous tissue. However, Luigetti et al.,^20^ based on CSF results, ruled out this direct invasion of virus. Patients with severe respiratory distress of COVID‐19 showed more neurological complications, which supports the reports of intracranial arterial stroke in hospitalized patients, even those treated with intravenous immunoglobulin.^21^ Emerging evidence has suggested a low frequency of COVID‐19 in children; inflammatory conditions in children with COVID‐19 receiving chimeric antigen receptor T‐cell therapy influence both CNS and PNS, giving rise to neurological manifestations.^27^ Patients who experienced delirium and other serious neurological complications require long‐term stay in ICU with mechanical ventilation and higher doses of sedatives and neuroleptics than other asymptomatic patients, resulting in delayed discharge from the hospital.^18^

Depression, anxiety, delirium, paranoia, hallucination, suicidal trial, affect lability, and psychomotor agitation were psychiatric symptoms observed in studied patients; depression (41.50%) and anxiety (20.85%) were the most frequently occurring symptoms. Delirium, a serious mental disability that diminish consciousness, showed an inflammatory origin following SARS‐CoV‐2 infection.^18^ CNS disorders are characterized by seizure/convulsion, stroke, coma, meningism, isolated cranial nerve affection, dysexecutive function, orthostatic hypertension, orthostatic intolerance, ataxia, acute fulminant cerebral edema, and other cranial nerve impairments. Seizure/convulsion, stroke, and coma are the most frequently symptoms reported in studies; however, isolated cranial nerve affection (26.49%) and dysexecutive function (71.43%) were found separately by an article as the most examined symptoms. Increased blood coagulation and cytokine levels, as well as the employment of immune cell components have been exhibited to cause tissue destruction, ultimately leading to stroke in patients with SARS‐CoV‐2. Karadaş et al.^8^, ^10^ and Chougar et al.^8^, ^10^ believe that this rare complication is rooted in the virus invasion to blood vessels. Other prognosis for stroke includes the elevated levels of leukocytes, D‐dimer, CRP, lactate dehydrogenase, ferritin in serum,^4^, ^45^ and low count of lymphocyte.^43^ Seizure is not a common neurological symptom of SARS‐CoV‐2, and the increased seizure reported in our investigation, according to Khedr et al.’s^26^ study, may have a link to the neurology specialization admission of cases with neurological manifestations. However, the occurrence of seizures in patients with COVID‐19 has been denoted to be associated with metabolic changes, fever,^10^ or the existence of tumors in CNS.^22^ Cerebral venous thrombosis, transient ischemic attacks, intracranial hemorrhage, CVD, vasculopathy, and hypoxic/ischemic brain injury are the main symptoms appeared in cerebrovascular disorders, which CVD (29.64%), vasculopathy (18.52%), and hypoxic/ischemic brain injury (10.72%) were the major symptoms. Cerebrovascular endotheliitis and the simultaneous presence of several disorders such as hypertension and diabetes were associated with increased tissue neurotropism SARS‐CoV‐2 predisposition of patients with COVID‐19 to cerebrovascular disorder by ACE2 expression enhancement. SARS‐CoV‐2 disrupts blood pressure homeostasis by blocking ACE2, a factor involved in the reduction of blood pressure.^26^ Encephalopathy, encephalitis, transverse myelitis, myelitis, meningoencephalitis, GBS and acute CNS infection/ADEM are inflammatory disorders of CNS. Encephalopathy, encephalitis, and GBS were extensively studied in articles, and encephalopathy reported as the most inflammatory disorders related to CNS following COVID‐19. Iltaf et al.^4^, ^23^ and Studart‐Neto et al.^4^, ^23^ speculated that encephalitis and encephalopathy are connected with the increased secretion of proinflammatory cytokines (e.g. IL‐2, ‐6, ‐7, TNF‐α, IFN‐γ) and antioxidant compounds (e.g., free radicals). GBS is a myelin‐destroying disorder induced by the immune system and causes muscular weakness, likely due to the similarity of the structure of SARS‐CoV‐2 epitopes with myelin proteins, which results in an autoimmune reaction.^10^, ^26^ Although being a rare complication, GBS is mostly caused by a wide range of viral and bacterial microorganisms through gastrointestinal infections such as Campylobacter and in rare cases, by respiratory infections such as SARS‐COV‐2.^4^, ^12^ In PNS disorders, the most frequent symptoms were anosmia/hyposmia, ageusia/dysageusia, and visual impairment. Other manifestations included auditory dysfunction, peripheral vestibular syndrome, sensory symptoms, myasthenia gravis, and relapse/attack of RR‐MS and RLS. Anosmia and ageusia disorders can be interpreted by the fact that the SARS‐CoV‐2 uses ACE2 receptors in the olfactory tissue to access the nerves.^22^, ^26^ Myalgia, myopathy, movement disorder, myositis, dysarthria/dysphagia, rhabdomyolysis, facial droop, decerebrate posturing, and skeletal muscle injury are known among neuromuscular disorders, which myalgia, as most the studied neuromuscular disorders, is considered to have inflammatory origin^46^ and related to the high levels of lactate dehydrogenase and creatine kinase.^43^ The latter may be relevant to the presence of ACE2 in muscles; however, this surmise needs further investigation.

Non‐specific neurological manifestation encompass headache, dizziness, weakness, fatigue, impaired consciousness/confusion, syncope, neuropathy, altered mental status, neuralgia, sleep impairment, daytime sleepiness, bifurcation in voice, balance disorder, numbness/paresthesia, vertigo, reduced reflexes, hemiparesis/hemiplegia, quadriplegia, peripheral facial palsy, hemineglect, and focal neurological deficit. Among these symptoms, headache, dizziness, impaired consciousness/confusion, and altered mental status were the most studied variables, while reduced reflexes, sleep impairment, daytime sleepiness, fatigue, dizziness, weakness, and headache showed high occurrence among patients with COVID‐19. Headache, as one of the frequent non‐specific manifestations, has reflected less association with mortality due to the fact that patients with headaches have less symptoms related to increased cytokine storm, such as D‐dimer, ferritin etc. Moreover, these patients had a history with lower blood pressure, as well as cardiovascular and nerve diseases. There is a direct relationship between headache and inflammation caused by COVID‐19 and high levels of IL‐6 as a marker of pain.^47^

One of the significant findings of the present study was the mortality rate (29.1%) due to neurological complications of COVID‐19. Other complications of COVID‐19 have been described as respiratory and non‐respiratory complications, which there is no doubt that respiratory complications have more severe outcome, as stated by Vakili and co‐workers.^48^ However, non‐respiratory complications are less prevalent but are more common in terms of mortality and can induce more risky situation for patients. According to LaRovere et al.’s study, neurological complications are not very serious; of 12% of patients who showed neurological complications, 66% developed death.^28^ Obviously, the neurological complications of COVID‐19 are associated with increased in‐hospital mortality of patients, which this statement is in agreement with Chou et al.’s^49^ cohort study. In‐hospital death of patients with COVID‐19 is also related to sex (i.e., males), diabetes mellitus, history of chronic pulmonary disease, increasing age, D‐dimer, body mass index, sequential organ failure assessment scores, and so forth. The number of neurological manifestations such as stroke and altered mentation (*p* < 0.05) in COVID‐19 patients may be a high‐risk factor for hospital‐related mortality.^31^, ^50^ Considering the reduced mortality rate of patients who were admitted in ICU, dexamethasone showed effective outcomes due to its inhibitory activity against immune response, which is essential for the inflammatory conditions. However, these corticosteroids induce neurological manifestations in long‐term administration.^51^ The mortality rate was higher in patients with the in vitro symptoms of COVID‐19 who suffered from neurological complications. This finding agrees with the study conducted by Frontera et al.^19^ who highlighted that patients with stroke are more prone to death than those who have stroke but not COVID‐19, which is attributed to prolonged hospitalization.

Mortality rate of neurological complications vary from country to country, owing to the age of patients and the level of access to treatment.^51^ According to WHO recent report (14 July 2021), the case fatality rate of patients infected with COVID‐19 was estimated to be 2.15%. Since no study has compared the mortality rate of respiratory and neurological complications, the mortality rate of COVID‐19‐related neurological complications (29.1%) obtained by the present study were compared with that of severe respiratory complications of COVID‐19 (39%) reported in Hasan et al.’s^52^ survey. They portrayed that the mortality rate of neurological complications in COVID‐19 patients was not higher than respiratory complications in these patients. Of note, serious neurological manifestations in patients with COVID‐19 cannot be ignored, and the mortality from respiratory complications of this disease varies in different countries, as reported to be 13% and 73% for Germany and Poland, respectively.^52^

There were some limitations for the current study. First, in addition to the bias of publications, which is common among almost systematic reviews, there were few heterogeneities in the prevalence of neurological complications and the number of studies. Second, inclusion criteria and excluding case reports and non‐English studies may lead to dismiss specific neurological complications. Third, since the COVID‐19 pandemic has not yet over, the results and underlying mechanisms of this disease would certainly be much more impressive than we expect. Unlike some systematic reviews that reviewed the majority of studies conducted in China, this work attempted to avoid the risk of bias by reviewing articles from different countries. Moreover, as there are no reports of neurological mortality from COVID‐19 and no data on the overall prevalence of neurological complications, this review has notable superiority over other similar studies in providing comprehensive information during a wider period of time.

## 5 CONCLUSION

The results of this systematic review and meta‐analysis showed that neurological complications may commonly occur in patients with COVID‐19. Neurological complications in COVID‐19 patients can arise from virus invasion to nervous tissues and post reactions such as immune‐related damage. Our study reported the prevalence of neurological complications and mortality rate of 23% and 29.1%, respectively. It indicated that headache, confusion, and fatigue were the most common neurological complications caused by COVID‐19. Taken together, patients with COVID‐19 who indicated neurological symptoms should be taken seriously and should receive early treatment to prevent undesirable events.

## CONFLICT OF INTEREST

The authors declare that they have no conflict of interest.

## AUTHOR CONTRIBUTIONS

Marzie Mahdizade Ari, Mohamad Hosein Mohamadi, Negar Shadab Mehr, Sajjad Abbasimoghaddam, Amirhosein Shekartabar, Mohsen Heidary, and Saeed Khoshnood contributed to revising and final approval of the version to be published. All authors agreed and confirmed the manuscript for publication.

## Supporting information

Table S1

## Data Availability

All the data in this review are included in the article.
