# Supplementary material for: Neurological manifestations in patients with COVID‐19: A systematic review and meta‐analysis
Source: J Clin Lab Anal. 2022 Apr 6;36(5):e24403. doi: 10.1002/jcla.24403 (PMC9102520; doi:10.1002/jcla.24403)
Supplement: Supplementary file 1 — Table S1 [file JCLA-36-e24403-s001.docx]

***Supplement table 1. Quality assessment for validation studies on Neurological manifestations in patients with COVID-19***

| First author | Was the sample representative of the target population? | Were study participants recruited in an appropriate way? | Was the sample size adequate? | Were the study subjects and the setting described in detail? | Was the data analysis conducted with sufficient coverage of the identified sample? | Were objective, standard criteria used for the measurement of the condition? | Was the condition measured reliably? | Are all important confounding factors/subgroups/differences identified and accounted for? | Were subpopulations identified using objective criteria? | Was there appropriate statistical analysis? |
| --- | --- | --- | --- | --- | --- | --- | --- | --- | --- | --- |
| Helms | Yes | No | Yes | No | Yes | Yes | Yes | No | No | Yes |
| Karadaş | Yes | No | Yes | No | Yes | Yes | Yes | No | No | Yes |
| Frontera | Yes | No | No | Yes | Yes | Yes | Yes | No | No | Yes |
| Luigetti | No | Yes | Yes | Yes | Yes | Yes | Yes | No | No | Yes |
| Kandemirli | Yes | Yes | Yes | No | Yes | Yes | Yes | No | No | Yes |
| Sandoval | Yes | Yes | Yes | Yes | Yes | Yes | Yes | No | Yes | Yes |
| Studart-neto | Yes | Yes | Yes | Yes | Yes | Yes | Yes | Yes | Yes | Yes |
| Xiong | No | No | Yes | Yes | Yes | Yes | Yes | No | No | Yes |
| Liguori | Yes | Yes | Yes | Yes | Yes | Yes | Yes | No | No | Yes |
| Scullen | No | No | No | Yes | Yes | Yes | Yes | Yes | Yes | Yes |
| Iltaf-Sr | Yes | Yes | Yes | No | Yes | Yes | Yes | No | No | Yes |
| Khedr | Yes | Yes | Yes | Yes | Yes | Yes | Yes | No | No | Yes |
| Chougar | Yes | No | No | Yes | Yes | Yes | Yes | Yes | Yes | Yes |
| Nersesjan | Yes | No | Yes | No | Yes | Yes | Yes | No | No | Yes |
| Abled-Mannan | Yes | Yes | No | Yes | Yes | Yes | Yes | Yes | Yes | Yes |
| LaRovere |  |  |  |  |  |  |  |  |  |  |
| Rifino | Yes | Yes | Yes | Yes | Yes | Yes | Yes | No | Yes | Yes |
| Mao | Yes | Yes | Yes | Yes | Yes | Yes | Yes | Yes | Yes | Yes |
| Eskandar | No | No | Yes | Yes | Yes | Yes | Yes | No | No | Yes |
| Shekhar | Yes | Yes | Yes | Yes | Yes | Yes | Yes | No | No | Yes |
| Helms | Yes | No | Yes | Yes | Yes | Yes | Yes | Yes | Yes | Yes |
| Karadaş | No | No | Yes | Yes | Yes | Yes | Yes | No | Yes | Yes |
| Frontera | No | No | Yes | Yes | Yes | Yes | Yes | No | Yes | Yes |
| Luigetti | Yes | Yes | Yes | No | Yes | Yes | Yes | No | No | Yes |
| Scullen | Yes | No | Yes | Yes | Yes | Yes | Yes | Yes | Yes | Yes |
| Iltaf-Sr | No | No | Yes | Yes | Yes | Yes | Yes | No | Yes | Yes |
| Khedr | No | No | Yes | Yes | Yes | Yes | Yes | No | Yes | Yes |
| Chougar | Yes | Yes | Yes | No | Yes | Yes | Yes | No | No | Yes |
| Nersesjan | Yes | Yes | Yes | No | Yes | Yes | Yes | No | No | Yes |
| Abled-Mannan | Yes | Yes | Yes | Yes | Yes | Yes | Yes | No | No | Yes |
| LaRovere | Yes | No | Yes | Yes | Yes | Yes | Yes | Yes | Yes | Yes |
| Rifino | No | No | Yes | Yes | Yes | Yes | Yes | No | Yes | Yes |
| Mao | No | No | Yes | Yes | Yes | Yes | Yes | No | Yes | Yes |
| Eskandar | Yes | Yes | Yes | No | Yes | Yes | Yes | No | No | Yes |
| Shekhar | Yes | No | Yes | Yes | Yes | Yes | Yes | Yes | Yes | Yes |
